# Supplementary material for: A high latitude Gondwanan species of the Late Devonian tristichopterid Hyneria (Osteichthyes: Sarcopterygii)
Source: PLoS One. 2023 Feb 22;18(2):e0281333. doi: 10.1371/journal.pone.0281333 (PMC9946258; doi:10.1371/journal.pone.0281333)
Supplement: S1 File — (PDF) [file pone.0281333.s001.pdf]

ALBANY MUSEUM

Devonian Ecosystems Project
87 Beaufort Street
Makhanda, Eastern Cape
South Africa
12 December 2022

Re: Use of painting of portion of Waterloo Farm lagerstätte (Fig. 13) in PLOS article on *Hyneria*.

Dear PLOS editors

This letter serves to confirm that copyright to the painting utilised as Figure 13 in 'A high latitude Gondwanan species of the Late Devonian tristichopterid *Hyneria* (Osteichthyes: Sarcopterygii)' resides with myself. The work was commissioned from illustrator Maggie Newman by myself (in my capacity as the sole proprietor of Rob Gess Consulting). I was responsible for providing the scientific input into the painting, it was funded through my consultancy and the final product is in my possession.

I am happy, for the advancement of scientific knowledge, for the painting to be made freely available online, and (assuming acknowledgement of its source) for it to be used without further condition by third parties.

Your sincerely

R.W. Gess

Dr Robert W. Gess
