# Supplementary material for: A high latitude Gondwanan species of the Late Devonian tristichopterid Hyneria (Osteichthyes: Sarcopterygii)
Source: PLoS One. 2023 Feb 22;18(2):e0281333. doi: 10.1371/journal.pone.0281333 (PMC9946258; doi:10.1371/journal.pone.0281333)
Supplement: S2 File — (PDF) [file pone.0281333.s002.pdf]

Science JOURNALS AAAS

AAAS Author License to Publish Policy

OUR PHILOSOPHY

The American Association for the Advancement of Science (AAAS), the non-profit publisher of the *Science* family of journals, recognizes the importance of openness in research as well as the increasingly global nature of the scientific enterprise. The broad public communication of peer-reviewed research, and information-sharing within the scientific community, are essential to advancing science in the service of society.

This is why AAAS makes all of its peer-reviewed research content freely available with registration via the *Science* Web site twelve months after publication. AAAS also makes any articles of urgent public-health importance freely and immediately available to the public, and it participates in various efforts to provide no-cost research content to the world's poorest countries.

As part of our mission, AAAS seeks to enhance communication among scientists, engineers, and the public, worldwide. Toward that end AAAS' license to publish agreement enables authors to use and share their manuscript in a variety of ways.

FURTHER DISSEMINATION

Authors are encouraged to further disseminate the accepted version of their manuscript, for release on the following schedule:

- Immediately following publication of the work by AAAS:
  - To the author's personal website
  - To the author's institution's archival database
- Six months following publication of the work by AAAS:
  - To the funder's* designated repository, if any

*On a trial basis, AAAS is allowing cOAlition S-funded authors to place a CC BY license (or a CC BY-ND license on their accepted manuscript. This policy will apply to any research submitted to the Science family subscription journals on or after 01 January, 2021 that was funded by those cOAlition S organizations that indicated adoption of the Plan S Rights Retention Strategy. (See here: https://www.coalition-s.org/plan-s-funders-implementation/.)

Additionally, authors and their institutions are encouraged—without further permission from AAAS—to use the final, published version of their work for educational purposes such as presentations, class handouts, dissertations, or photocopies for colleagues, so long as that use is not commercial in nature.
Further details about the AAAS author license to publish agreement appear below.

OUR LICENSE TO PUBLISH

1. **Authors grant to AAAS an exclusive license to publish, not a copyright transfer.**
   In consideration of publication by AAAS in one of its *Science* journals, the author grants to AAAS an exclusive license to publish and to reproduce, adapt, and distribute the work as well as related supplemental content as broadly as possible, in various languages and forms, worldwide. Ownership of the copyright will remain with the author. AAAS will be the title holder for purposes of registration.

2. **Authors can immediately use final works for non-profit purposes—no permission needed.**
The author retains the non-exclusive right to use the final, published version of the work, immediately after it is made public by AAAS and without further permission, for educational and other non-commercial purposes, for example, in print collections of the author's own writings; in the author's thesis or dissertation (for electronic deposits, however, only the accepted manuscript should be used); oral presentations by the author; educational courses taught by the author or offered by the author's institution; and distribution of photocopies by the author to colleagues for non-commercial purposes (not for further re-distribution). Figures from the final, published version of the work also can be used, with attribution, in future works by the author, including journal articles, book chapters, presentation slides, and posters.

3. **Authors also can share the final published version through the AAAS "referrer linking service."**
Authors may take advantage of the AAAS referrer linking service in order to provide free access to their final, published work via their personal or institutional Web site. AAAS will provide authors with one free referrer link per article. Information on the service will be e-mailed to the corresponding author the week following publication by AAAS.

4. **Authors can immediately post the accepted work to their personal or institutional archive.**
Authors also retain the right and are encouraged to post the accepted version of their manuscript to their personal Web site or institutional repository, immediately following publication by AAAS. (The accepted version is the paper that was accepted for publication by AAAS, including changes resulting from peer review, but prior to AAAS copyediting.) In such cases, authors must include a hyperlink to the final version published on the *Science* Web site, along with an explanatory note that includes the full citation, as follows: "This is the author's version of the work. It is posted here by permission of the AAAS for personal use, not for redistribution. The definitive version was published in *[Science* Journal Title] on [Volume number and date], DOI: [insert DOI number].".

5. **Authors can release the accepted work to designated repositories, as required by funders.**
Authors of research articles, reports, reviews, or technical comments can release the accepted version of their work to PubMedCentral or another designated repository no sooner than six months after publication by AAAS. The accepted work can be *submitted* to the funder's repository immediately upon publication by AAAS, but authors must ensure that it is not scheduled for posting or public release for at least six months.

6. **Plan S funded authors.**
On a trial basis, authors of research that was funded by those cOAlition S organizations that have implemented a Plan S aligned OA policy may, upon publication by AAAS, distribute their accepted manuscript under a CC BY license (or a CC BY-ND license, if permitted by the funder). This trial is effective starting with appliable papers submitted to the Science family subscription journals on or after 01 January, 2021.

7. **Authors and their institutions can use the accepted work for non-profit research purposes.**
   Information-sharing is a key to scientific progress. Authors and their institutions therefore can use the accepted version of manuscripts for non-commercial research purposes, including sharing results with colleagues, or for replication or adaptation of the work so long as such use is not for any commercial advantage or exploitation. No further permission is needed from AAAS. Authors and their institutions cannot license the work, except to AAAS.

8. **Authors pledge that the work is original, true, and not previously published.**
   The author(s) warrant that the work is original, that all facts are true and accurate, that it has not been published elsewhere, and does not infringe on any copyright, proprietary, or personal right of any third party. If the work contains materials owned or controlled by a third party, the author must certify that s/he has obtained permission for its use by attaching documentation to the License to Publish form and by clearly attributing the source within the text of the manuscript.

9. **University or institutional restrictions require a waiver.**
   If university or other institutional restrictions might limit the authors' ability to grant to AAAS any of the rights described in AAAS's license, they must obtain an approved waiver from their institution.

10. **Publication by AAAS will require a signed copy of AAAS's License to Publish form.**
    This License to Publish must be signed and returned to AAAS before a manuscript can be accepted for publication. Each author must sign the form. If the contribution is owned by the author's employer, an authorized representative must sign the form.

11. **Authors must sign AAAS's form even if the Work was created under U.S. Government Contract.**
    The AAAS recognizes the U.S. Government's non-exclusive rights to use the work for non-commercial, governmental purposes where such rights are established in the grant or contract.

12. **Authors employed by the U.S. Government should sign in the appropriate section of AAAS's form.**
    The area designated for U.S. Government employees confirms that the Work was written as part of your official duties as an employee of the U.S. Government, and therefore the Work is in the public domain.
