# Supplementary material for: A high latitude Gondwanan species of the Late Devonian tristichopterid Hyneria (Osteichthyes: Sarcopterygii)
Source: PLoS One. 2023 Feb 22;18(2):e0281333. doi: 10.1371/journal.pone.0281333 (PMC9946258; doi:10.1371/journal.pone.0281333)
Supplement: Attachment — Submitted filename: Response to Reviewers.docx [file pone.0281333.s004.docx]

**PONE-D-22-29333: Response to Reviewers**

We thank the reviewers and academic editor for their constructive comments. Here are our responses to both:

**Specific name:** We note the ICZN recommendation but do not entirely agree with it. Linnaeus himself named the silver bream (a cyprinid fish) *Abramis bjoerkna*, using the Swedish vernacular name "björkna" as the specific name. Furthermore, there is a strong current trend for using unmodified vernacular names: *Tiktaalik* (Inuktituk) and *Guiyu* (Mandarin Chinese) are familiar recent examples. We will stick with *udlezinye* but have noted in the text that it is an apposition.

**Style requirements:** We have applied the style templates in the links provided by you.

**Inclusivity questionnaire:** Completed and uploaded.

**Funding information / Financial Disclosure:** We have removed the funding-related text from the Acknowledgements. The online Financial Disclosure statement is correct. In the Current Funding Sources List, the GENUS award to Rob Gess is missing because this funding initiative (which falls under the National Research Foundation) cannot be found in your data base.

**Ethics statement:** We have included an ethics statement in the Methods section.

**Figure 1 (maps):** Figure 1A has previously been published in *PLoS ONE* (Gess & Trinajstic 2017, doi: 10.1371/journal.pone.0173169) and should thus not present a problem. Figure 1B is reproduced from Gess & Ahlberg 2018 (*Science* **360**, doi: 10.1126/science.aaq1645). *Science's* copyright policy (uploaded) states specifically that the author retains copyright and is free to re-use the image without restrictions as long as it is attributed. The figure legend has been modified to cite the sources of the images.

**Figure 13:** The copyright of this image (a painting by Maggie Newman) resides with the first author, who commissioned it from her. A letter explaining its status and granting you permission to reproduce the image has been uploaded. The figure legend has been modified to state the copyright.

**Registration in ZooBank:** This has been carried out and the new LSIDs have been added to the manuscript in accordance with your instructions.

**Comments of Reviewer #1:**

1) It is evident for two reasons that the shape differences we observe between *Hyneria lindae* and *H. udlezinye* are taxonomically significant rather than just allometric size effects. Firstly, large individuals of the two species are of approximately the same size, a shown by this comparison of postparietal shields:

Secondly, in the case of the subopercular (arguably the most morphologically distinctive bone of *H. udlezinye*), small and large examples show the same dorsoventrally shallow shape, as demonstrated in our figure 8E-F and G-H (compare the scale bars!). We have amended the text to emphasise that these suboperculars are similar in shape even though they differ in size.

2) The preservation of the dentary fangs (and other large teeth) in the *H. udlezinye* material is really interesting: they are typically black, whereas the bone is silver, and their outlines are often 'wavy' as well as incomplete. It looks like they were completely demineralised with only the organic matrix of the dentine being preserved. However, we are confident that the dentary fangs are larger than in *H. lindae*, for the simple reason that the preserved parts are of noticeably larger dimensions. The only uncertainty concerns exactly how tall they were, but there is no positive reason to believe that their proportions were different from those of *H. lindae* or many other Devonian-Carboniferous sarcopterygians. We have not amended the text.

**Comments of Reviewer #2:**

We appreciate this positive assessment of the value of our paper.
